# Microarray analysis reveals a potential role of LncRNAs expression in cardiac cell proliferation

**DOI:** 10.1186/s12861-016-0139-4

**Published:** 2016-11-18

**Authors:** Jue Wang, Zhimin Geng, Jiakan Weng, Longjie Shen, Ming Li, Xueli Cai, Chengchao Sun, Maoping Chu

**Affiliations:** 1 Department of Cardiac Surgery, the First Affiliated Hospital of Wenzhou Medical University, Nanbaixiang, Shangcaicun, Wenzhou, 325000 Zhejiang Province People’s Republic of China; 2 Children’s Heart Center, the Second Affiliated Hospital & Yuying Children’s Hospital, Institute of Cardiovascular Development and Translational Medicine, Wenzhou Medical University, No. 109, Xueyuan Road, Wenzhou, 325000 Zhejiang Province People’s Republic of China; 3 Tianjin Childrens’ Hospital, Tianjin, People’s Republic of China; 4 Department of Transplantation, the First Affiliated Hospital of Wenzhou Medical University, Wenzhou, Zhejiang Province People’s Republic of China; 5 Cardiac Regeneration Research Institute, Wenzhou Medical University, Wenzhou, Zhejiang Province People’s Republic of China; 6 Department of Cardiology, the First Affiliated Hospital of Wenzhou Medical University, Wenzhou, Zhejiang Province People’s Republic of China

**Keywords:** Long non-coding RNA, Microarray, Human heart, Cardiac cell proliferation

## Abstract

**Background:**

Long non-coding RNAs (LncRNAs) have been identified to play important roles in epigenetic processes that underpin organogenesis. However, the role of LncRNAs in the regulation of transition from fetal to adult life of human heart has not been evaluated.

**Methods:**

Immunofiuorescent staining was used to determine the extent of cardiac cell proliferation. Human LncRNA microarrays were applied to define gene expression signatures of the fetal (13–17 weeks of gestation, *n* = 4) and adult hearts (30–40 years old, *n* = 4). Pathway analysis was performed to predict the function of differentially expressed mRNAs (DEM). DEM related to cell proliferation were selected to construct a lncRNA-mRNA co-expression network. Eight lncRNAs were confirmed by quantificational real-time polymerase chain reaction (*n* = 6).

**Results:**

Cardiac cell proliferation was significant in the fetal heart. Two thousand six hundred six lncRNAs and 3079 mRNAs were found to be differentially expressed. Cell cycle was the most enriched pathway in down-regulated genes in the adult heart. Eight lncRNAs (*RP11-119 F7.5, AX747860, HBBP1, LINC00304, TPTE2P6, AC034193.5, XLOC_006934* and *AL833346*) were predicted to play a central role in cardiac cell proliferation.

**Conclusions:**

We discovered a profile of lncRNAs differentially expressed between the human fetal and adult heart. Several meaningful lncRNAs involved in cardiac cell proliferation were disclosed.

**Electronic supplementary material:**

The online version of this article (doi:10.1186/s12861-016-0139-4) contains supplementary material, which is available to authorized users.

## Background

The heart is the first-formed organ during human embryogenesis. Approximately 10 weeks after fertilization, four important processes, namely, looping, trabeculation, septation and myocardial compaction, have been completed in the human fetal heart. Through the remaining pregnancy period, the myocardium undergoes specialization into specific cardiac tissues. Previous studies have elucidated that the architecture of human myocardium changes from isotropic to anisotropic during development [1]. During the fetal period, the major source of energy is glucose for the heart, which is different from a full utilization of fatty acid oxidation in the adult period [2]. Moreover, cardiomyocyte proliferation is evident during fetal life, but the proliferation capacity decreases in the adult heart [3]. During the transition from fetal to adult life of the heart, sophisticated regulatory networks are required to adapt to diverse physiological and pathophysiological responses. Recently, it has become evident that LncRNAs may in fact play major roles in most aspects of gene regulation, especially in the epigenetic processes that underpin organogenesis [4].

LncRNAs are long non-coding RNAs, which can function as either primary or spliced transcripts, and they are independent of the currently known classes of small RNAs (micro RNAs, piwi-interacting RNAs, and others) and exclude classic housekeeping families of RNAs (such as tRNAs and rRNAs) [5]. In recent years, accumulating evidence has indicated that lncRNAs are involved in a series of vital biological processes, such as serving as precursors for smaller RNAs and controlling the cell cycle [6], cell apoptosis [7], brain and testis development as well [8, 9]. It has been reported that lncRNAs can impact gene expression at multiple levels (e.g., transcriptional and post-transcriptional control, epigenetic regulation). Moreover, lncRNAs have been found to be able to regulate the expressions of proximal and distal protein-coding genes through cis- and trans-acting mechanisms [10]. Until now, many lncRNAs have been identified in humans, which prompt the building of human lncRNA database providing expression profiles of lncRNAs [11].

Currently, several lncRNAs have been shown to exert critical roles in cardiac development and regeneration, such as lncRNA-*Braveheart* [12], lncRNA-*Fendrr* [13] and lncRNA-*ST8SIA3* [14]. However, most of the literature on cardiac profiling is based on animal models, such as mice. As there is a great difference between human heart and animal heart, and lncRNA sequences generally exhibit low conservation across species [15], it is requisite to determine the lncRNA profile in human fetal and adult heart and find key lncRNAs that regulate the transition from fetal to adult life of the heart. To this end, we have used high-throughput microarray lncRNA screening to investigate differentially expressed lncRNAs and mRNAs between human fetal hearts and adult hearts. As a result, pathways analysis revealed that the down-regulated genes in the adult group were mainly related to cell proliferation. Particular lncRNAs were predicted to play important roles in cardiac cell proliferation.

## Methods

### Tissue collection

The fetal cardiac tissues used in this study were collected from patients undergoing prostaglandin induction of labor for termination of pregnancy due to unwanted pregnancy. Six fetal hearts were obtained, and the gestational ages were between 13 and 17 weeks. Adult cardiac tissues were collected from six unused organ donor hearts aged between 30 and 40 years old. The heart was carefully separated from the rest of the thoracic content with sterilized surgical instruments. After dissection, the heart was investigated and confirmed to have normal cardiac anatomy and then washed with PBS buffer (Ambion, USA). Myocardial samples were taken from the left ventricle and immediately stored in liquid nitrogen.

### Immunofiuorescent staining

The fetal and adult heart tissues were fixed in 4% paraformaldehyde. Sections of myocardial tissues were mounted onto glass slides. For immunofiuorescent staining, the sections were permeabilized in 0.3% Triton X-100 in PBS solution for 5 min, followed by blocking in 5% goat serum plus 5% donkey serum for 1 h. Then the sections were stained with the primary antibodies overnight at 4 °C. The primary antibodies were Mouse anti-cardiac Troponin T antibody (cTnT, Abcam) and Rabbit anti-Ki-67 antibody (Abcam), which diluted in blocking solution (1:200). The appropriate secondary antibodies conjugated to DyLight-594 and Fitc-488 (Abcam) were added to the tissues for 1 h at room temperature and the tissues were washed with PBST (PBS buffer with 0.05% Tween 20) for 10 min and PBS for 5 min. The cell nuclei were counter-stained with 4, 6-diamidino-2-phenylindole (DAPI, Beyotime) for 2 min and then covered with 70% (v/v) glycerol. After mounted with glass cover slips, the immunofiuorescent staining was captured under a laser confocal fluorescence microscope. (Nikon, A1).

### RNA extraction

RNA was extracted from frozen myocardial tissue using Trizol reagent (Invitrogen Life Technologies, Carlsbad, CA, USA). The quantification and quality of the extracted RNA were measured using a Nano Drop ND-1000 spectrophotometer. The high purity of the isolated RNA, as indicated by A260/280 ≥ 1.90, was confirmed before microarray and quantificational real-time polymerase chain reaction (qRT-PCR) experiments.

### Microarray analysis

RNA samples were analyzed through the microarray. An Arraystar Human LncRNA Microarray v 3.0 (8 × 60 K, Arraystar) was used, which can detect 30,586 lncRNAs and 26,109 protein-coding transcripts. Briefly, rRNA was removed from total RNA, and then, mRNA was obtained (mRNA-ONLY™ Eukaryotic mRNA Isolation Kit, Epicentre). The random priming method was utilized to amplify each sample, and mRNA was transcribed into fluorescent cRNAs without 3’bias. Labeled cRNAs were hybridized to the Human LncRNA Microarray. An Agilent Microarray Scanner (Agilent p/n G2565BA) was used to scan the microarray after the slides were washed. The acquired array images were analyzed using Agilent Feature Extraction software (version 11.0.1.1). After performing quantile normalization, lncRNAs and mRNAs for which at least four of the eight samples had present or marginal flags (“All Targets Value”) were chosen for further data analysis using the GeneSpring GX v11.5.1 software package (Agilent Technologies).

### Pathway analysis

DEM were uploaded into the Kyoto Encyclopedia of Genes and Genomes database (KEGG, http://www.genome.ad.jp/kegg/). Pathway analysis was applied to analyze the potential pathways that DEM involved.

### Co-expression network construction

To predict the key lncRNAs that function in the proliferation of cardiac cells, lncRNA-mRNA co-expression network was constructed on the basis of the correlation between the differentially expressed lncRNAs and mRNAs that related to cell proliferation. Pearson’s correlation coefficient was calculated to measure the gene co-expression, and the absolute value ≥ 0.99975 and *P*-value < 0.001 were considered to be strong correlated between lncRNAs and mRNAs. In the network, a node represents a gene and two correlated genes are connected with an edge, with solid line representing positive correlation and dotted line representing negative correlation. Square nodes represent lncRNAs, while circular nodes represent mRNAs. Red nodes are symbols of down-regulated genes and blue ones are up-regulated genes in the adult heart. Degree is used to describe the number of genes related to the key lncRNAs. The larger the node is, the higher the degree would be.

### qRT-PCR validation assay

The SYBR green method was used to perform qRT - PCR in an Applied Biosystems 7300 Sequence Detection System (ABI 7300 SDS; Foster City, CA, USA). The reaction conditions of PCR included a denaturation step of 10 min at 95 °C and 40 cycles of 15 s at 95 °C and 1 min at 60 °C. The gene expression levels were normalized to the housekeeping gene *GAPDH*, and all samples were measured in triplicate. The relative expression levels of the genes were calculated using the 2 ^– ΔΔCt^ method.

### Statistical analysis

All of the data are shown as the mean ± standard deviation. The statistical significance of differentially expressed lncRNAs between the fetal heart and the adult heart, were analyzed using Student’s t-test in SPSS 16.0 (SPSS, Chicago, IL, USA). Fisher’s exact test was used to examine the significance of the pathway correlated to the conditions, *P* < 0.05 and FDR < 0.05 were considered statistically significant. FDR was used to correct the *P* values.

## Results

### Cardiac cell proliferation in fetal and adult hearts

**Fig. 1:**
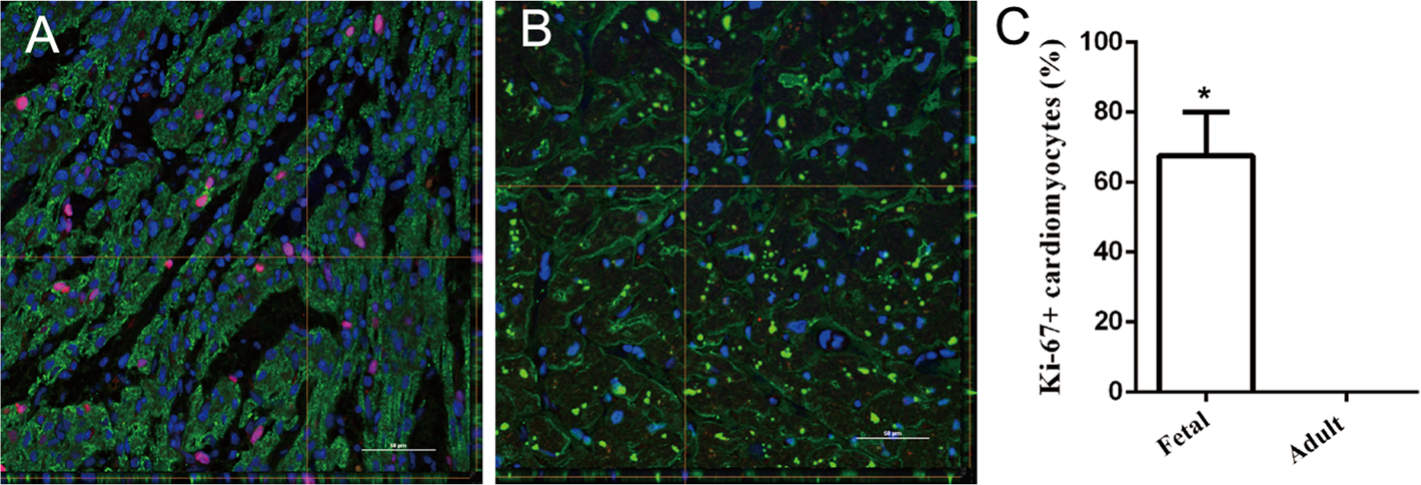
Extent of cardiomyocyte proliferation in fetal and adult hearts. Immunofluorescent staining shows the proliferating Ki-67+ cardiac cell in fetal (**a**) and adult heart (**b**) respectively. Scale bar: 50 μm. The yellow lines indicate the position within the image of projections given in the lower and right. Percentage of Ki-67 positive cardiomyocyte in all positive cells was shown respectively (**c**). Data are expressed as the mean ± standard deviation (SD) of three independent experiments (*P < 0.01)

We firstly used two antibodies (Ki-67 and cardiac Troponin T (cTnT)) to stain myocardium sections of both fetal and adult hearts to determine the extent of cardiac cell proliferation. Ki-67 has been reported to be detected in proliferating cells at all active phases of cell cycle while cTnT is a cardiomyocyte marker that expresses in both cycling and non-cycling cells. Here, Ki-67 stained cells were observed in fetal heart (Fig. 1a), not in adult heart (Fig. 1b), proving that the cardiac cell proliferation only occurred in fetal heart. Ki-67 positive cardiomyocytes and all Ki-67 positive cells were quantified. Percentage of Ki-67 positive cardiomyocytes in fetal myocardium is 67.73% while adult myocardium is zero (Fig. 1c).

### Differentially expressed LncRNAs and mRNAs

**Fig. 2:**
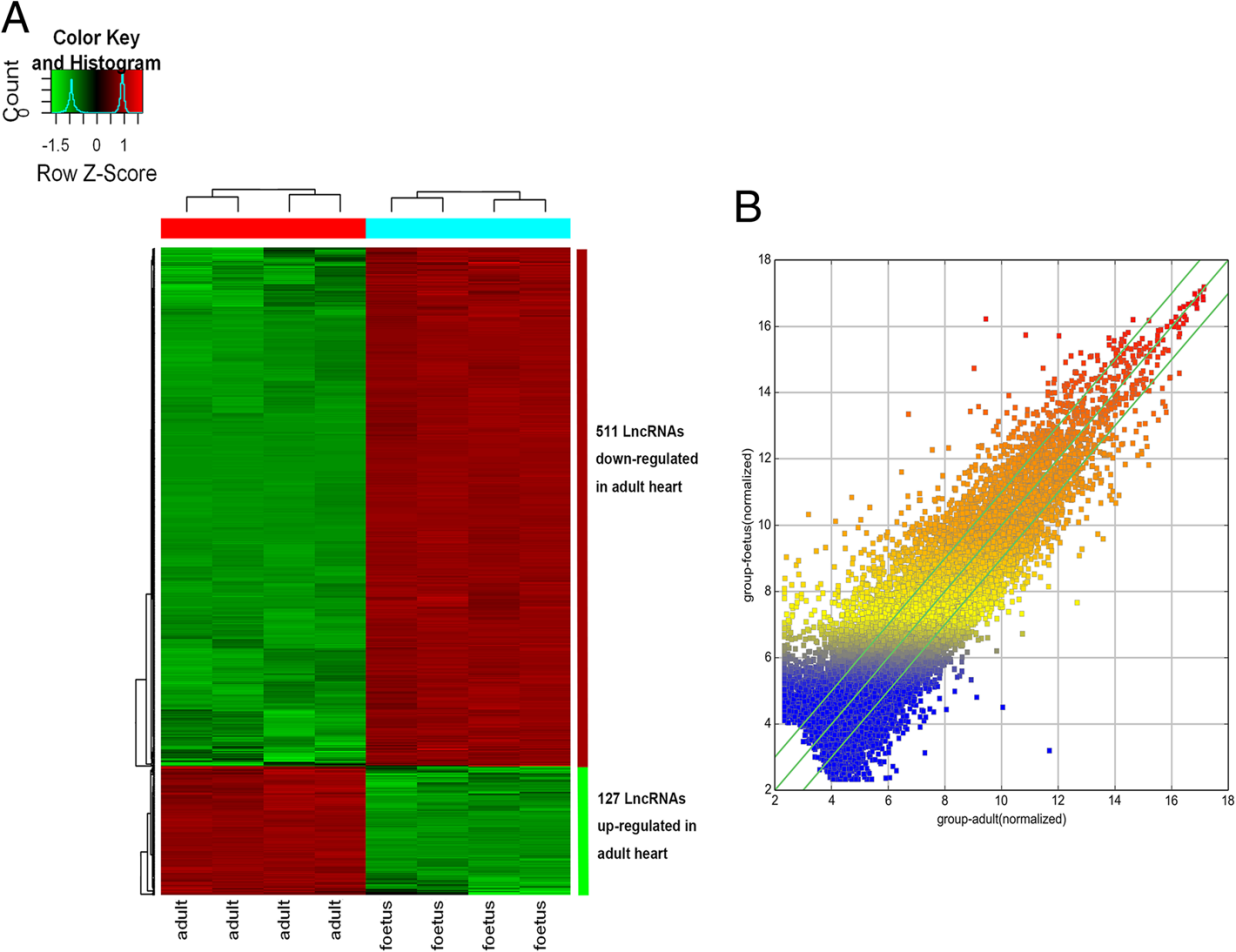
Differential expression of lncRNAs between fetal and adult heart. Hierarchical clustering analysis shows differential expression of LncRNAs (FC ≥ 5) using a heat map (**a**). ‘Red’ indicates higher expression, whereas ‘blue’ indicates lower expression. The bar code represents the color scale of log2 transformed values. The scatterplot (**b**) is a visualization used for assessing the variation between the chips. The X and Y axes in the scatterplot represent the normalized signal values of each group (log2 scaled). The green lines represent FC (FC = 2.0) lines

LncRNA expression profiling data showed that 18,137 lncRNAs and 20,363 protein-coding RNAs were expressed using microarray analysis. Thus, we evaluated the absolute ratio of normalized intensities in paired samples (adult-to-fetal ratio, A/F). Two thousand six hundred six lncRNAs were found to be differentially expressed (fold change, FC ≥ 2) between two groups. One thousand three hundred forty five lncRNAs were up-regulated while 1261 were down-regulated in the adult group, compared with those in the fetal group (Additional file 1). A total of 357 lncRNAs were found to be significantly differentially expressed with a FC of five, among which 285 lncRNAs were down-regulated and 72 lncRNAs were up-regulated in the adult group, compared with the fetal group (Table 1). The top ten down-regulated and top ten up-regulated lncRNAs were listed in Table 2. Hierarchical clustering analysis has shown significantly up-regulated and down-regulated lncRNAs with FC ≥ 5 (Fig. 2a). A scatterplot was a visualization used to assess the variation between the chips (Fig. 2b). Additionally, 1446 mRNAs were down-regulated, and 1633 were up-regulated in the adult group compared with the fetal group (Additional file 2).

**Table 1:** Number of differentially expressed LncRNAs

LncRNAs	FC 2 - 5	FC ≥ 5	FC ≥ 10	Total
Down	894	285	82	1261
Up	1259	72	14	1345

**Table 2:** Top 10 up-regulated and down-regulated LncRNAs

Down-regulated LncRNAs			Up-regulated LncRNAs		
Sequence name	Gene symbol	FC	Sequence name	Gene symbol	FC
*ENST00000568019*	*PWRN1*	141.7670014	*ENST00000581502*	*RP11-76 K13.3*	357.5387068
*ENST00000449258*	*RP11-406O23.2*	99.982081	*NR_027242*	*SSTR5-AS1*	31.9642591
*ENST00000581798*	*CTD-3096 M3.2*	99.3375837	*TCONS_00007953*	*XLOC_003422*	15.8564648
*ENST00000425771*	*GAS5*	70.4895431	*uc001loz.3*	*BC040735*	12.592759
*ENST00000382864*	*EGFEM1P*	68.5222621	*ENST00000523269*	*MIR143HG*	12.5208372
*ENST00000573315*	*LINC00514*	66.1058412	*ENST00000514473*	*AE000661.37*	12.2791441
*ENST00000483846*	*EGFEM1P*	65.7167316	*ENST00000556777*	*AE000661.37*	12.1201584
*ENST00000554841*	*RP1-261D10.2*	63.4768112	*TCONS_00018898*	*XLOC_008763*	11.9494669
*ENST00000445310*	*KCNQ5-IT1*	54.1499847	*TCONS_00013650*	*XLOC_006306*	11.6920115
*ENST00000463978*	*RP11-71H9.1*	53.5463368	*ENST00000512263*	*RP11-161D15.1*	11.3482059
*TCONS_00015370*	*XLOC_006934*	52.5315592	*ENST00000425802*	*RP11-91A18.4*	10.44422

### Pathway enrichment analysis

**Fig. 3:**
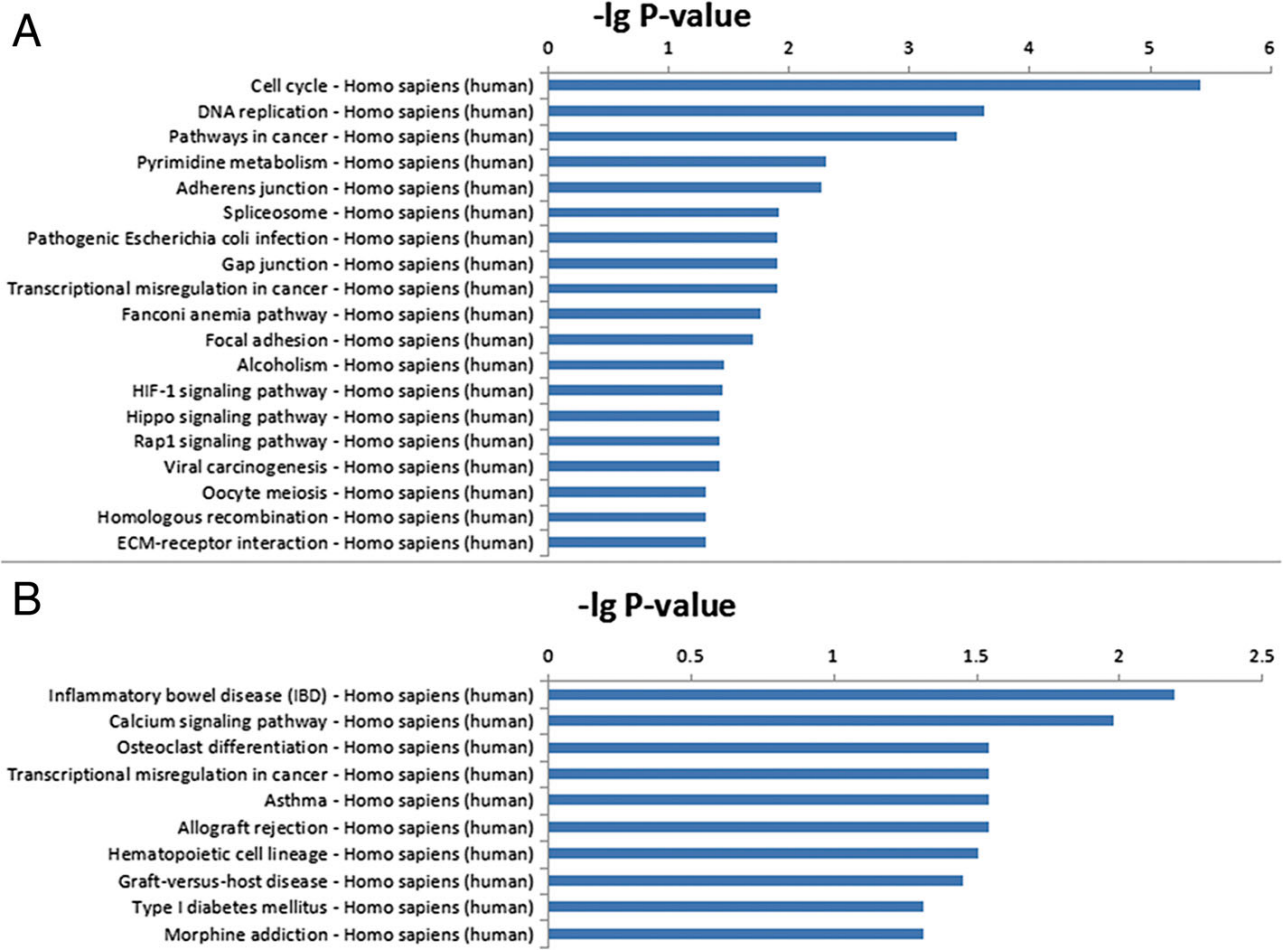
Pathway analysis. The pathways that exhibited significant differences between fetal and adult hearts are listed. A shows the down-regulated genes pathways and B the up-regulated genes pathways in adult group. Higher -lg *P*-value indicates higher significance level

Pathway enrichment analysis indicated that down-regulated genes in the adult group were involved in 19 pathways, while the up-regulated genes were involved in 10 pathways. We have used -lg *P*-value to describe significance level of the pathway enrichment (Fig. 3). The enriched pathways including “cell cycle pathway”, “DNA replication”, “p53 signaling pathway” and “Hippo signaling pathway”, in down-regulated genes in the adult group, may have an important effect on cell proliferation. Among these pathways, the cell cycle pathway was the most enriched pathway related to the down-regulated genes.

The pathways involved in junction formation in cardiomyocyte such as “adherents junction” and “gap junction were also enriched in the down-regulated genes. In addition, calcium signaling pathway, which relates to the development of the cardiac conduction system, was represented in the up-regulated genes. Pathways relating to the immune system were flagged up in up-regulated genes. These included allograft rejection and graft-versus-host disease. The differentially expressed genes involved in the above pathways will provide a foundation for future study on the underlying mechanism of the changes that occur during the second trimester of heart development.

### LncRNA-mRNA co-expression network

**Fig. 4:**
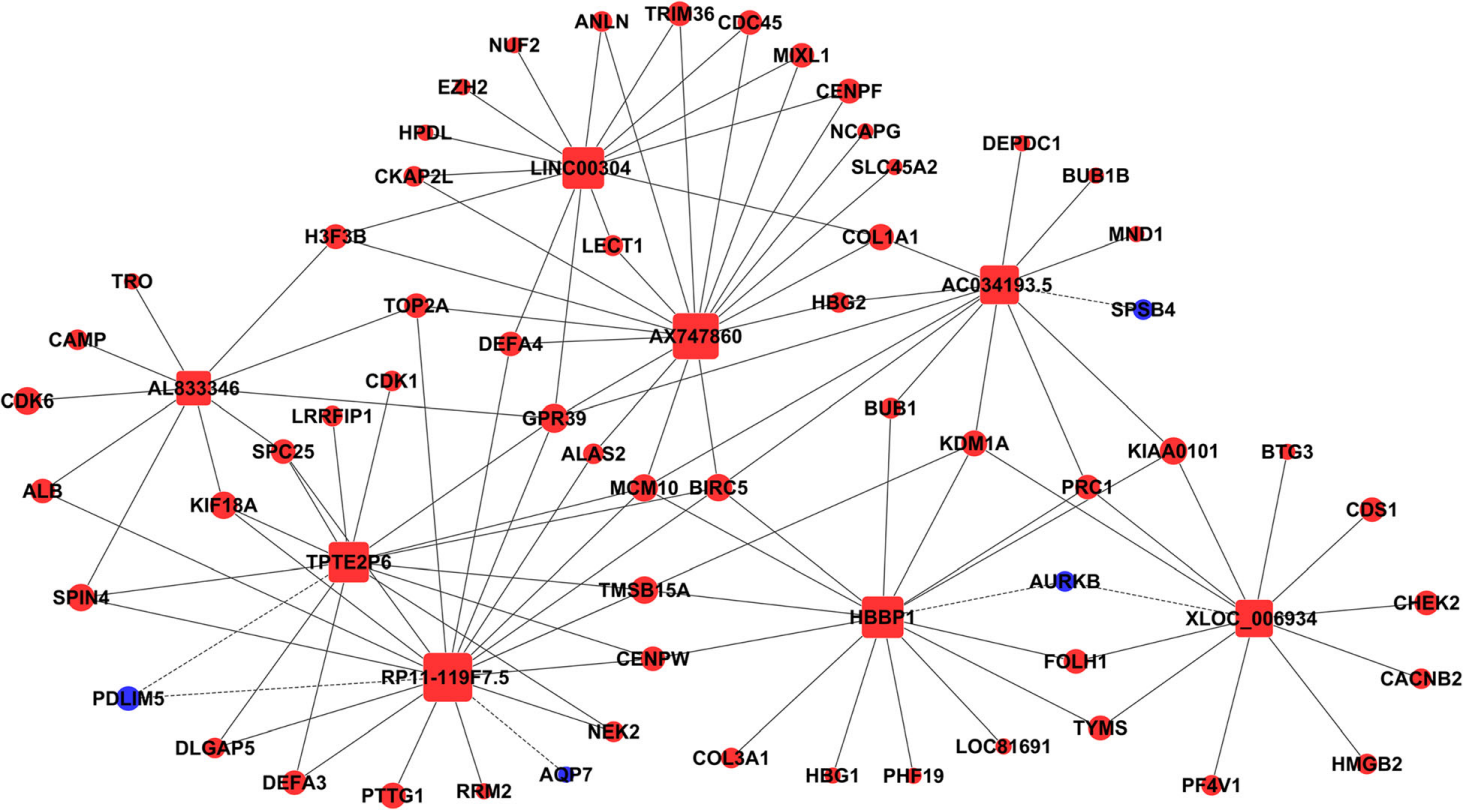
Establishment of lncRNA-mRNA co-expression network. Nodes represent genes and an edge is used to connect two correlated genes. Solid lines represent positive correlation and dotted lines negative correlation. Blue nodes represent up-regulated genes and red nodes represent down-regulated genes in adult heart. Square nodes represent lncRNAs, while circular nodes represent mRNAs. The larger the node is, the higher the degree would be

Previously published studies have demonstrated that lncRNAs play a critical role in gene transcription [16]. Here, we constructed a lncRNA-mRNA co-expression network to investigate how the differential expression of the lncRNAs involved cardiac cell proliferation. DEM involved in the pathways associated with cell proliferation were selected to establish a lncRNA-mRNA co-expression network. 242 pairs of lncRNAs and mRNAs were identified to be coexpressed (Additional file 3), with that 220 pairs were positively correlated and 22 pairs negatively correlated. *XLOC_009300* - *MMP1*, *AX747860* - *ANLN*, and *RP11-119 F7.5* - *DEFA3* were the highest positive correlated lncRNA-mRNA pairs, and these lncRNAs may promote the expression of the related mRNA. *RP11-119 F7.5* - *PDLIM5*, *RP11-71H9.*1 - *ISCU* and *RP5-902P8.10* - *RPIA* were the highest negative, this suggests that these lncRNAs may inhibit the expression of the related mRNA. LncRNA *RP11-119 F7.5*, *AX747860*, *HBBP1*, *LINC00304*, *TPTE2P6*, *AC034193.5, XLOC_006934* and *AL833346*, which were shown to have the highest degree, may play core roles in the co-expression network (Fig. 4). These lncRNA-mRNA pairs may play the crucial roles in the co-expression network.

### Microarray validation by qRT-PCR

**Fig. 5:**
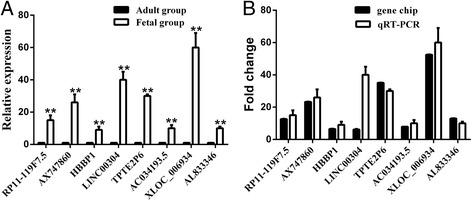
qRT-PCR validation of differential expressions of LncRNAs. **a** Eight LncRNAs confirmed by qRT-PCR show to have significant changes between fetal and adult groups. Data are expressed as the mean ± standard deviation (SD) of three independent experiments (*P < 0.01). **b** qRT-PCR patterns of seven LncRNAs except for LINC00304 are completely consistent with those of microarray data. Data of gene chip group are expressed as the mean ± standard deviation (SD) (*n* = 4, normalized data). The Y-axis represents FCs (A/F)

The eight focused lncRNAs, which were identified to be closely related with cardiac cell proliferation through lncRNA-mRNA co-expression network construction, were selected to verify their expression level and the microarray results using qRT-PCR with samples (*n* = 6/group). The qRT-PCR analysis showed that the eight lncRNAs changed significantly between the fetal and adult groups (Fig. 5a), and the expression tendency was confirmed to be concordant with the microarray data (Fig. 5b).

## Discussion

In the current study, high-throughput microarray LncRNAs screening was utilized to identify differentially expressed lncRNAs between human fetal and adult hearts. Pathways relating to cell proliferation were most enriched in the down- regulated genes and pathways relating to immune system were most enriched in the up- regulated genes in the adult heart. Particular lncRNAs were predicted to play important roles in cardiac cell proliferation by the establishment lncRNA-mRNA co-expression network.

Cardiomyocytes proliferation is important during heart development as it is required for normal heart morphogenesis and increases the heart size [17]. Although proliferation level of mammalian cardiomyocyte is high during fetal period, it starts to decrease in postnatal stages [18]. It has been found that the heart loses its regenerative ability which exists in the fetal heart when the cardiomyocyte proliferation declines in the adult heart [19, 20]. Presently, we have demonstrated that cardiac cell proliferation was significant in the second trimester and cannot be detected in the adult heart by immunofiuorescent staining. Pathway analysis was performed on DEM and it revealed that down-regulated genes in the adult heart were mainly featured by the annotation “cell cycle”. This finding suggests proliferative phenotype of cardiac cell in the second trimester, which collaborates with the immunohistological observations. These results are almost consistent with findings in mouse [21]. It has been reported that Hippo-deficient embryos had overgrown hearts with elevated cardiomyocyte proliferation in developing mouse heart [22]. However, our results showed that Hippo pathway was enriched in the down-regulated genes, which was different from the discovery in the mouse heart. Cardiac specific knockout of LATS2 and WW45 in mice resulted in over-grown hearts with elevated cardiomyocyte proliferation [22], but these two genes were not regulated in our result. In addition, YAP1, which is one of the key genes in Hippo pathway found in mouse heart [23, 24], was downregulated in the adult heart with FC of 5.59. This implied that YAP may also impede cardiomyocyte proliferation in the adult human heart.

What is more, allograft rejection pathway and graft-versus-host disease pathway were involved significantly in the up-regulated genes in the adult heart. This result suggests that the activation of immune response in the second trimester, which was also found in the transition from 2- to 13-day-old mouse hearts [25].

Recently, increasing evidence has indicated that lncRNAs play significant roles in the proliferation of various cell types [26]. However, very few studies have been conducted on the potential functions of lncRNAs in cardiac cell proliferation. In the current study, 638 lncRNAs were found to be significantly differentially expressed (FC ≥ 5.0) between the fetal and adult hearts. Most of the discovered lncRNAs were not functionally characterized, so lncRNA-mRNA co-expression network was constructed to predict the key lncRNAs that related to cell proliferation. LncRNA *RP11-119 F7.5, AX747860, HBBP1, LINC00304, TPTE2P6, AC034193.5, XLOC_006934* and *AL833346*, which were shown to have the highest degree, may play core roles in the co-expression network. The results imply that the regulation of these genes may affect cardiac cell proliferation through regulating the expression of their corresponding mRNAs.

Several significantly changed lncRNAs were predicted to be closely related to organ development and cardiac function. LncRNA *ENST00000425771*, a 242 nt lncRNA transcribed from a gene called growth arrest-specific 5 (*GAS5*), is a lncRNA located on chromosome 1. *GAS5* can act as a riborepressor of the glucocorticoid receptor by binding to the DNA-binding domain of the glucocorticoid receptor [27], and glucocorticoids have been demonstrated to be critical for cardiac development [28]. Recently, increasing evidence has indicated that *GAS5* plays a critical role in the proliferation of cancer cells [29, 30]. Therefore, we infer that the down-regulated lncRNA *ENST00000425771* with a FC of 70.4895431 may function in the change in proliferation capacity of cardiomyocytes during cardiac development. LncRNA *NR_038439* is a 1450 nt antisense transcript from the protein coding gene *CACNA1G*, and the FC of down-regulated lncRNA *NR_038439* was 29.0475756. Previous findings show that *CACNA1G*, which is related to the T-type calcium currents, is mainly expressed in young and adult hearts [31, 32]. This finding is consistent with our microarray data showing up-regulated expression of *CACNA1G* (FC = 29) in the adult heart compared with the fetal heart. The expression of *CACNA1G* may be involved in hormone secretion [31].

## Conclusion

In summary, we discovered for the first time a profile of lncRNAs differentially expressed between the human fetal and adult heart. Several meaningful lncRNAs involved in cardiac cell proliferation were disclosed. Although the mechanisms of the discovered lncRNAs in cardiac cell proliferation remain to be elucidated, we hope our novel discovery will lead to more studies that will determine its function.

In our future study, we will focus on the function of the above lncRNAs on several kinds of cardiac cells, such as cardiomyocytes, vascular endothelial cells, smooth muscle cells and cardiac fibroblasts, and hope to find the key factors that are able to promote cardiac cells proliferation.

## Additional files

Additional file 1: Differentially Expressed LncRNAs. (XLS 2.36 mb)
Additional file 2: Differentially Expressed mRNAs. (XLS 4.45 mb)
Additional file 3: Coexpresses lncRNA-mRNA pairs. (PDF 126 kb)

## Abbreviations

A/F: Adult-to-fetal ratio
DEM: Differentially expressed mRNAs
FC: Fold change
GAS5: Growth arrest-specific 5
LncRNA: Long non-coding RNA
