# Supplementary material for: Microarray analysis reveals a potential role of LncRNAs expression in cardiac cell proliferation
Source: BMC Dev Biol. 2016 Nov 18;16:41. doi: 10.1186/s12861-016-0139-4 (PMC5116129; doi:10.1186/s12861-016-0139-4)
Supplement: Additional file 3: — Coexpresses lncRNA-mRNA pairs. (PDF 126 kb) [file 12861_2016_139_MOESM3_ESM.pdf]

Coexpresses lncRNA-mRNA pairs

Gene1	type1	Gene2	type2	correlation_va	p_value	regulation
RP1-240B8.3	noncoding	BAIAP2L2	protein_coding	-0.99993794	5.98E-13	negative
RP11-474I16.8	noncoding	MIF	protein_coding	-0.9999317	7.96E-13	negative
RP11-119F7.5	noncoding	PDLIM5	protein_coding	-0.99992995	8.59E-13	negative
WHAMMP2	noncoding	CECR1	protein_coding	-0.99992593	1.02E-12	negative
RP11-71H9.1	noncoding	ISCU	protein_coding	-0.99992447	1.08E-12	negative
RP11-466A19.6	noncoding	MFI2	protein_coding	-0.99990669	2.03E-12	negative
XLOC_000026	noncoding	POLR1C	protein_coding	-0.9998928	3.08E-12	negative
RP11-448G15.3	noncoding	LILRB4	protein_coding	-0.99988775	3.54E-12	negative
OR2A9P	noncoding	MOGAT3	protein_coding	-0.99988267	4.04E-12	negative
XLOC_001718	noncoding	CCNB1	protein_coding	-0.99987601	4.76E-12	negative
HBBP1	noncoding	AURKB	protein_coding	-0.99987075	5.40E-12	negative
AK301968	noncoding	ADAMTSL5	protein_coding	-0.99986432	6.24E-12	negative
RP6-201G10.2	noncoding	CHRM1	protein_coding	-0.99985838	7.10E-12	negative
RP3-331H24.4	noncoding	LCORL	protein_coding	-0.99984339	9.60E-12	negative
XLOC_005099	noncoding	PTN	protein_coding	-0.99984003	1.02E-11	negative
XLOC_001718	noncoding	DMRTC1B	protein_coding	-0.99983909	1.04E-11	negative
RP11-881M11.4	noncoding	COL13A1	protein_coding	-0.99983798	1.06E-11	negative
RP11-1105O14.1	noncoding	C3orf58	protein_coding	-0.99983244	1.18E-11	negative
RP11-24M17.5	noncoding	PF4V1	protein_coding	-0.99982853	1.26E-11	negative
RP11-71H9.1	noncoding	KIAA0895L	protein_coding	-0.99982838	1.26E-11	negative
PMCHL2	noncoding	KIAA0895L	protein_coding	-0.99982578	1.32E-11	negative
RP3-331H24.4	noncoding	IRX2	protein_coding	-0.99982404	1.36E-11	negative
XLOC_010533	noncoding	CKS1B	protein_coding	-0.99982342	1.38E-11	negative
GOLGA2B	noncoding	CLDN9	protein_coding	-0.99982238	1.40E-11	negative
SCAND2	noncoding	FAM125A	protein_coding	-0.99981998	1.46E-11	negative
AP000462.3	noncoding	AGPAT2	protein_coding	-0.99981928	1.48E-11	negative
LOC650368	noncoding	HLA-DRB1	protein_coding	-0.99981623	1.55E-11	negative
RP11-24M17.5	noncoding	DLC1	protein_coding	-0.9998159	1.56E-11	negative
RP5-848E13.3	noncoding	GPC4	protein_coding	-0.99981522	1.58E-11	negative
RCAN3AS	noncoding	C2orf54	protein_coding	-0.99980944	1.73E-11	negative
LOC84989	noncoding	GRAMD1A	protein_coding	-0.99980716	1.79E-11	negative
LOC100272217	noncoding	HCFC1	protein_coding	-0.99980573	1.83E-11	negative
RP11-381O7.3	noncoding	HBA1	protein_coding	-0.9998032	1.91E-11	negative
RP11-841O20.2	noncoding	CENPN	protein_coding	0.999800663	1.98E-11	positive
LOC84989	noncoding	CKAP2L	protein_coding	0.999801733	1.95E-11	positive
LOC84989	noncoding	DLGAP5	protein_coding	0.999802319	1.93E-11	positive
RP11-613M10.6	noncoding	KIAA0101	protein_coding	0.999803317	1.90E-11	positive
RP5-902P8.10	noncoding	PIK3CD	protein_coding	0.999803341	1.90E-11	positive
RP1-86D1.3	noncoding	MFSD6L	protein_coding	0.999804329	1.87E-11	positive
LOC100505806	noncoding	DEFA3	protein_coding	0.999804386	1.87E-11	positive
XLOC_012317	noncoding	PTTG1	protein_coding	0.999804552	1.87E-11	positive
RP11-71H9.1	noncoding	FGB	protein_coding	0.999804944	1.86E-11	positive
XLOC_006934	noncoding	KDM1A	protein_coding	0.999804949	1.85E-11	positive
RP5-858B6.3	noncoding	ATP6V1G2	protein_coding	0.99980499	1.85E-11	positive
LOC100129617	noncoding	HPDL	protein_coding	0.99980525	1.85E-11	positive
CTC-367J11.1	noncoding	CCNG1	protein_coding	0.999805427	1.84E-11	positive
RP11-307C12.11	noncoding	FKBPL	protein_coding	0.999805556	1.84E-11	positive
RP11-155G14.5	noncoding	PLEK2	protein_coding	0.999806117	1.82E-11	positive
RP11-439E19.3	noncoding	GJA1	protein_coding	0.999806382	1.81E-11	positive
RP11-613M10.6	noncoding	CHEK2	protein_coding	0.999806838	1.80E-11	positive
RP1-86D1.3	noncoding	FAM69C	protein_coding	0.999807596	1.78E-11	positive
RP11-1114A5.4	noncoding	BEX2	protein_coding	0.999808071	1.77E-11	positive
RP11-389O22.1	noncoding	ACYP1	protein_coding	0.999808642	1.75E-11	positive
PLAC2	noncoding	CCNG1	protein_coding	0.999809205	1.74E-11	positive
LOC100506123	noncoding	CDK6	protein_coding	0.999809503	1.73E-11	positive

XLOC_006934	noncoding	KIAA0101	protein_coding	0.999809887	1.72E-11	positive
RP4-564F22.2	noncoding	GLIPR2	protein_coding	0.999810084	1.71E-11	positive
RP1-86D1.3	noncoding	C11orf82	protein_coding	0.999810418	1.70E-11	positive
LINC00304	noncoding	CKAP2L	protein_coding	0.999810572	1.70E-11	positive
AK094859	noncoding	ACVR2B	protein_coding	0.999810899	1.69E-11	positive
RPS27P25	noncoding	PPM1B	protein_coding	0.999810987	1.69E-11	positive
RP11-454P7.3	noncoding	HBZ	protein_coding	0.999811152	1.68E-11	positive
HHIP-AS1	noncoding	HBZ	protein_coding	0.999811318	1.68E-11	positive
XLOC_012829	noncoding	ADAMTSL5	protein_coding	0.999812169	1.66E-11	positive
LOC729970	noncoding	KIF14	protein_coding	0.999812459	1.65E-11	positive
XLOC_014103	noncoding	SPARC	protein_coding	0.999813359	1.63E-11	positive
RP11-434B12.1	noncoding	IFT52	protein_coding	0.999814336	1.60E-11	positive
TPTE2P6	noncoding	MCM10	protein_coding	0.999814489	1.60E-11	positive
RP11-368I23.2	noncoding	ARID5B	protein_coding	0.999814959	1.58E-11	positive
RP11-551L14.1	noncoding	KCTD15	protein_coding	0.99981526	1.58E-11	positive
XLOC_001743	noncoding	NUP62CL	protein_coding	0.999816108	1.55E-11	positive
RP11-280O1.2	noncoding	LRRN1	protein_coding	0.999816369	1.55E-11	positive
LINC00304	noncoding	DEFA4	protein_coding	0.999816377	1.55E-11	positive
PRKY	noncoding	HBG1	protein_coding	0.99981643	1.55E-11	positive
RP11-353N14.2	noncoding	RNASE2	protein_coding	0.99981652	1.54E-11	positive
XLOC_011166	noncoding	NHLRC2	protein_coding	0.999816569	1.54E-11	positive
RP11-196G18.23	noncoding	MBOAT2	protein_coding	0.999816906	1.53E-11	positive
RP11-1114A5.4	noncoding	C17orf96	protein_coding	0.999817024	1.53E-11	positive
RP11-199F6.4	noncoding	ENOSF1	protein_coding	0.999817039	1.53E-11	positive
RP11-75C9.1	noncoding	ITPA	protein_coding	0.999818457	1.50E-11	positive
CTD-2119F7.2	noncoding	AP1S2	protein_coding	0.99981906	1.48E-11	positive
RP11-500G22.2	noncoding	GLIPR2	protein_coding	0.999819201	1.48E-11	positive
MLLT4-AS1	noncoding	IL33	protein_coding	0.999819648	1.47E-11	positive
RP11-119F7.5	noncoding	DEFA4	protein_coding	0.999819894	1.46E-11	positive
XLOC_009300	noncoding	BPI	protein_coding	0.999820183	1.45E-11	positive
RP11-704J17.5	noncoding	NPPB	protein_coding	0.999820261	1.45E-11	positive
RP11-71H9.1	noncoding	LETMD1	protein_coding	0.999820917	1.44E-11	positive
LOC389023	noncoding	C8orf42	protein_coding	0.99982102	1.43E-11	positive
TUBA4B	noncoding	CRABP2	protein_coding	0.999821057	1.43E-11	positive
XLOC_006934	noncoding	CHEK2	protein_coding	0.999821939	1.41E-11	positive
XLOC_013181	noncoding	IFNGR2	protein_coding	0.999822177	1.41E-11	positive
RP11-106E15.1	noncoding	CKAP2L	protein_coding	0.999822679	1.39E-11	positive
LOC100507217	noncoding	LAMB1	protein_coding	0.99982302	1.39E-11	positive
RP11-841O20.2	noncoding	LNX2	protein_coding	0.999823417	1.38E-11	positive
LOC84989	noncoding	CAMP	protein_coding	0.999823713	1.37E-11	positive
CTC-340A15.2	noncoding	CDK19	protein_coding	0.999823933	1.36E-11	positive
SMA4	noncoding	RNASEH2A	protein_coding	0.99982414	1.36E-11	positive
TPTE2P6	noncoding	BIRC5	protein_coding	0.999824342	1.35E-11	positive
HBBP1	noncoding	CENPW	protein_coding	0.999824352	1.35E-11	positive
XLOC_001718	noncoding	ARID1A	protein_coding	0.999824435	1.35E-11	positive
LRRC37A4	noncoding	CENPN	protein_coding	0.999824705	1.35E-11	positive
RP11-287A8.6	noncoding	NT5DC2	protein_coding	0.999824994	1.34E-11	positive
RP11-503E24.2	noncoding	HPX	protein_coding	0.999825302	1.33E-11	positive
RP11-298O21.5	noncoding	PORCN	protein_coding	0.999825549	1.33E-11	positive
RP11-106E15.1	noncoding	HIST1H3G	protein_coding	0.999825686	1.32E-11	positive
RP11-71H9.1	noncoding	KIF14	protein_coding	0.999826151	1.31E-11	positive
RP11-199F6.4	noncoding	POLR1D	protein_coding	0.999826685	1.30E-11	positive
TSPY26P	noncoding	TMSB15A	protein_coding	0.999826874	1.30E-11	positive
LINC00304	noncoding	ANLN	protein_coding	0.999827545	1.28E-11	positive
RP1-315G1.3	noncoding	CENPN	protein_coding	0.999828085	1.27E-11	positive
CTD-3080F16.3	noncoding	CAMP	protein_coding	0.999828127	1.27E-11	positive

TPTE2P6	noncoding	NEK2	protein_coding	0.99982835	1.26E-11	positive
RP11-831F12.2	noncoding	7-Mar	protein_coding	0.999828639	1.26E-11	positive
HBBP1	noncoding	COL3A1	protein_coding	0.999829399	1.24E-11	positive
TPTE2P6	noncoding	SPIN4	protein_coding	0.999829443	1.24E-11	positive
TMPO-AS1	noncoding	COL1A1	protein_coding	0.999829505	1.24E-11	positive
TSPY26P	noncoding	KIAA0101	protein_coding	0.999830009	1.23E-11	positive
RP11-206L10.9	noncoding	FNDC4	protein_coding	0.999830264	1.22E-11	positive
SCARNA9	noncoding	NOTCH2NL	protein_coding	0.999830346	1.22E-11	positive
RP11-368I23.2	noncoding	ACYP1	protein_coding	0.999830375	1.22E-11	positive
RP11-406O23.2	noncoding	CDK6	protein_coding	0.9998305	1.22E-11	positive
RP11-199F6.4	noncoding	C8orf48	protein_coding	0.999831878	1.19E-11	positive
RP3-522D1.1	noncoding	MBOAT2	protein_coding	0.999832433	1.18E-11	positive
RP11-119F7.5	noncoding	BIRC5	protein_coding	0.999832639	1.17E-11	positive
BANCR	noncoding	ADAMTSL1	protein_coding	0.999833008	1.16E-11	positive
KRT18P55	noncoding	FRZB	protein_coding	0.999833015	1.16E-11	positive
TSPY26P	noncoding	CACNB2	protein_coding	0.999834292	1.14E-11	positive
RP3-522D1.1	noncoding	GLIPR2	protein_coding	0.999834332	1.14E-11	positive
LINC00304	noncoding	H3F3B	protein_coding	0.999835003	1.12E-11	positive
SCARNA9	noncoding	KCNMB4	protein_coding	0.999835362	1.12E-11	positive
C17orf76-AS1	noncoding	ACVR2B	protein_coding	0.999835624	1.11E-11	positive
RP11-119F7.5	noncoding	ALB	protein_coding	0.999835722	1.11E-11	positive
LOC100506123	noncoding	ARID5B	protein_coding	0.999835913	1.10E-11	positive
RP11-613M10.6	noncoding	DLC1	protein_coding	0.999836027	1.10E-11	positive
C6orf147	noncoding	BEX2	protein_coding	0.999836476	1.09E-11	positive
RP11-119F7.5	noncoding	NEK2	protein_coding	0.999836597	1.09E-11	positive
KRT18P55	noncoding	CAMP	protein_coding	0.999836609	1.09E-11	positive
LRRC37A4P	noncoding	CACNB2	protein_coding	0.999836934	1.08E-11	positive
LINC00222	noncoding	FAM196B	protein_coding	0.999837638	1.07E-11	positive
LINC00304	noncoding	COL1A1	protein_coding	0.999837853	1.07E-11	positive
RP5-848E13.3	noncoding	ROBO2	protein_coding	0.999837865	1.07E-11	positive
TSSC2	noncoding	KRTDAP	protein_coding	0.999837893	1.06E-11	positive
RP5-991G20.4	noncoding	POU3F2	protein_coding	0.999838347	1.06E-11	positive
RP11-323F5.2	noncoding	BOD1	protein_coding	0.999839108	1.04E-11	positive
PLAC2	noncoding	MFSD6L	protein_coding	0.999839843	1.03E-11	positive
XLOC_006934	noncoding	BTG3	protein_coding	0.999839999	1.02E-11	positive
XLOC_010933	noncoding	SRSF9	protein_coding	0.999840474	1.01E-11	positive
UBE2Q2P3	noncoding	TSSK3	protein_coding	0.99984052	1.01E-11	positive
RP11-119F7.5	noncoding	MCM10	protein_coding	0.999840932	1.01E-11	positive
CR936711	noncoding	ARID5B	protein_coding	0.999841294	9.99E-12	positive
RP11-119F7.5	noncoding	DLGAP5	protein_coding	0.999841706	9.91E-12	positive
NOS2P1	noncoding	AGPAT4	protein_coding	0.999842402	9.78E-12	positive
TMPO-AS1	noncoding	MMP1	protein_coding	0.999844203	9.45E-12	positive
RP11-71H9.1	noncoding	CENPH	protein_coding	0.999844237	9.45E-12	positive
RP11-196G18.23	noncoding	OIP5	protein_coding	0.999845028	9.30E-12	positive
RP11-589P10.5	noncoding	RAB40AL	protein_coding	0.999845611	9.20E-12	positive
RP11-69I8.3	noncoding	OR2B6	protein_coding	0.999846973	8.96E-12	positive
RP11-368I23.2	noncoding	BEX1	protein_coding	0.999847868	8.80E-12	positive
TTC28-AS1	noncoding	KIDINS220	protein_coding	0.999848343	8.72E-12	positive
ITCH-IT1	noncoding	FAM43A	protein_coding	0.999849938	8.45E-12	positive
RP11-406O23.2	noncoding	ACYP1	protein_coding	0.999850029	8.43E-12	positive
XLOC_000564	noncoding	POMT1	protein_coding	0.999850101	8.42E-12	positive
RP11-1114A5.4	noncoding	CDC42EP3	protein_coding	0.999850918	8.28E-12	positive
RP11-82O19.2	noncoding	NOVA1	protein_coding	0.999853201	7.91E-12	positive
XLOC_007703	noncoding	C17orf72	protein_coding	0.999854825	7.65E-12	positive
RP11-73E17.2	noncoding	GOLIM4	protein_coding	0.999855183	7.59E-12	positive
OPCML-IT1	noncoding	GTSE1	protein_coding	0.999855331	7.57E-12	positive

RP11-448G15.3	noncoding	NDC80	protein_coding	0.999855442	7.55E-12	positive
SND1-IT1	noncoding	CCNB1IP1	protein_coding	0.999857519	7.23E-12	positive
SNORA33	noncoding	SGK1	protein_coding	0.999858086	7.14E-12	positive
C6orf147	noncoding	ACVR2B	protein_coding	0.999860217	6.83E-12	positive
LINC00501	noncoding	GART	protein_coding	0.999860256	6.82E-12	positive
RP11-14N7.2	noncoding	NLE1	protein_coding	0.999862215	6.54E-12	positive
TMPO-AS1	noncoding	CENPF	protein_coding	0.9998623	6.53E-12	positive
RP11-168C9.1	noncoding	PCDH18	protein_coding	0.999862596	6.48E-12	positive
BC040735	noncoding	BBC3	protein_coding	0.999862926	6.44E-12	positive
RP11-105C19.2	noncoding	KIF24	protein_coding	0.999863412	6.37E-12	positive
RP11-368I23.2	noncoding	CLK1	protein_coding	0.999864378	6.24E-12	positive
ZNF876P	noncoding	COIL	protein_coding	0.999864995	6.15E-12	positive
RP5-916O11.2	noncoding	MIXL1	protein_coding	0.999866709	5.92E-12	positive
RP1-315G1.3	noncoding	LNX2	protein_coding	0.999866763	5.91E-12	positive
XLOC_006934	noncoding	HMGB2	protein_coding	0.999868465	5.69E-12	positive
FAS	noncoding	CCNG1	protein_coding	0.999869438	5.56E-12	positive
XLOC_010933	noncoding	HADHA	protein_coding	0.999870669	5.41E-12	positive
TUBA4B	noncoding	GOLPH3	protein_coding	0.999873426	5.07E-12	positive
RP11-589P10.5	noncoding	C8orf48	protein_coding	0.999874461	4.95E-12	positive
RP11-37L2.1	noncoding	ASB10	protein_coding	0.999875859	4.78E-12	positive
RP11-466A19.6	noncoding	CHRM1	protein_coding	0.99987606	4.76E-12	positive
TPTE2P6	noncoding	DEFA3	protein_coding	0.999876306	4.73E-12	positive
TPTE2P6	noncoding	CENPW	protein_coding	0.999876435	4.72E-12	positive
RP11-54C4.1	noncoding	RALGAPA1	protein_coding	0.999878395	4.50E-12	positive
SCARNA9	noncoding	CACNB1	protein_coding	0.999879811	4.34E-12	positive
RP11-196G18.23	noncoding	BAMBI	protein_coding	0.999880126	4.31E-12	positive
AX747860	noncoding	ALAS2	protein_coding	0.999880416	4.27E-12	positive
LRRC37A4P	noncoding	BTG3	protein_coding	0.999881461	4.16E-12	positive
PDLIM3	noncoding	ADRB1	protein_coding	0.999882592	4.05E-12	positive
XLOC_009653	noncoding	GRK5	protein_coding	0.999884541	3.85E-12	positive
XLOC_010142	noncoding	PF4V1	protein_coding	0.999886543	3.65E-12	positive
XLOC_011177	noncoding	ST6GAL2	protein_coding	0.999886563	3.65E-12	positive
XLOC_002650	noncoding	HECW2	protein_coding	0.999888487	3.47E-12	positive
XLOC_006934	noncoding	FOLH1	protein_coding	0.999888842	3.43E-12	positive
RP11-495P10.2	noncoding	ACVR2B	protein_coding	0.999890669	3.27E-12	positive
RP11-627G18.3	noncoding	POLR1D	protein_coding	0.999891786	3.17E-12	positive
LDHA	noncoding	FZD1	protein_coding	0.999892254	3.13E-12	positive
LOC100132356	noncoding	ENPEP	protein_coding	0.999893894	2.99E-12	positive
RP11-500G22.2	noncoding	FAM69C	protein_coding	0.999895723	2.83E-12	positive
TMPO-AS1	noncoding	CDC45	protein_coding	0.999897183	2.72E-12	positive
LOC100506123	noncoding	KIDINS220	protein_coding	0.999897283	2.71E-12	positive
RP1-315G1.3	noncoding	NET1	protein_coding	0.999898363	2.62E-12	positive
RP11-199F6.4	noncoding	CKAP2	protein_coding	0.999899223	2.56E-12	positive
RP11-841O20.2	noncoding	C17orf96	protein_coding	0.999899368	2.55E-12	positive
RP4-564F22.2	noncoding	MBOAT2	protein_coding	0.999899623	2.53E-12	positive
RP11-75C9.1	noncoding	POC1A	protein_coding	0.999899637	2.53E-12	positive
PLAC2	noncoding	EIF2AK2	protein_coding	0.999900525	2.46E-12	positive
RP11-119F7.5	noncoding	CENPW	protein_coding	0.99990192	2.36E-12	positive
CLEC2D	noncoding	CDS1	protein_coding	0.999903378	2.26E-12	positive
LOC84989	noncoding	FRZB	protein_coding	0.999904866	2.15E-12	positive
RP11-199F6.4	noncoding	FAM200B	protein_coding	0.999905168	2.13E-12	positive
LINC00304	noncoding	CDC45	protein_coding	0.999905457	2.11E-12	positive
C17orf76-AS1	noncoding	BEX2	protein_coding	0.999905784	2.09E-12	positive
RP11-539G18.2	noncoding	COIL	protein_coding	0.9999086	1.91E-12	positive
RP11-119F7.5	noncoding	PTTG1	protein_coding	0.999909926	1.83E-12	positive
XLOC_006934	noncoding	TYMS	protein_coding	0.99990994	1.83E-12	positive

RP11-439E19.3	noncoding	HILPDA	protein_coding	0.999912187	1.69E-12	positive
RP11-119F7.5	noncoding	KIF18A	protein_coding	0.99991464	1.55E-12	positive
RP11-334C17.5	noncoding	CDC42EP3	protein_coding	0.999914777	1.55E-12	positive
RP11-334C17.5	noncoding	RAD51	protein_coding	0.999916175	1.47E-12	positive
TSPY26P	noncoding	TMEM45A	protein_coding	0.999918941	1.33E-12	positive
RP11-167J8.3	noncoding	DEPDC1	protein_coding	0.999919394	1.31E-12	positive
XLOC_014288	noncoding	SULT1E1	protein_coding	0.999920543	1.25E-12	positive
SMA5	noncoding	HPX	protein_coding	0.999920702	1.25E-12	positive
RP11-106E15.1	noncoding	EZH2	protein_coding	0.999923116	1.14E-12	positive
RP11-389O22.1	noncoding	MTF2	protein_coding	0.999924602	1.07E-12	positive
TMPO-AS1	noncoding	MIXL1	protein_coding	0.999925754	1.02E-12	positive
TPTE2P6	noncoding	SPC25	protein_coding	0.999927239	9.63E-13	positive
TSPY26P	noncoding	MTF2	protein_coding	0.99992742	9.56E-13	positive
RP11-71H9.1	noncoding	CDK1	protein_coding	0.999929482	8.77E-13	positive
TPTE2P6	noncoding	KIF18A	protein_coding	0.999930859	8.26E-13	positive
RP11-71H9.1	noncoding	LRRFIP1	protein_coding	0.999931946	7.88E-13	positive
LINC00501	noncoding	GMNN	protein_coding	0.999932228	7.78E-13	positive
RP11-199F6.4	noncoding	BBS7	protein_coding	0.999936125	6.51E-13	positive
RP5-916O11.2	noncoding	AUNIP	protein_coding	0.99993825	5.89E-13	positive
RP11-69I8.3	noncoding	FZD1	protein_coding	0.999944439	4.29E-13	positive
FLJ42875	noncoding	CACNB1	protein_coding	0.999944954	4.17E-13	positive
RP11-109A6.4	noncoding	ITSN2	protein_coding	0.999945183	4.12E-13	positive
LOC647946	noncoding	GTSE1	protein_coding	0.999948127	3.49E-13	positive
RP1-315G1.3	noncoding	C17orf96	protein_coding	0.99995246	2.69E-13	positive
RP11-119F7.5	noncoding	DEFA3	protein_coding	0.999958297	1.81E-13	positive
XLOC_011767	noncoding	EPX	protein_coding	0.99996081	1.51E-13	positive
AX747860	noncoding	ANLN	protein_coding	0.999961872	1.39E-13	positive
RP11-710F7.3	noncoding	MSL2	protein_coding	0.999970981	6.11E-14	positive
XLOC_006166	noncoding	CXorf27	protein_coding	0.999974687	4.04E-14	positive
XLOC_009300	noncoding	MMP1	protein_coding	0.999988275	4.00E-15	positive
SMCHD1	protein_coding	CTD-2384A14.2	noncoding	-0.99993967	5.49E-13	negative
PLSCR3	protein_coding	AK026379	noncoding	-0.99992437	1.08E-12	negative
RPIA	protein_coding	RP5-902P8.10	noncoding	-0.99992001	1.28E-12	negative
PDLIM5	protein_coding	KRT18P55	noncoding	-0.99991	1.82E-12	negative
SEPT10	protein_coding	AK055811	noncoding	-0.99990666	2.03E-12	negative
MOGAT3	protein_coding	CTD-2270L9.4	noncoding	-0.99989327	3.04E-12	negative
2-Mar	protein_coding	AK098438	noncoding	-0.99988804	3.51E-12	negative
LECT1	protein_coding	BC040735	noncoding	-0.99988492	3.81E-12	negative
SYNPO	protein_coding	RP5-848E13.3	noncoding	-0.99988467	3.83E-12	negative
ZNF579	protein_coding	CTD-2001E22.2	noncoding	-0.9998816	4.15E-12	negative
SEPT10	protein_coding	RP11-381O7.3	noncoding	-0.99987828	4.51E-12	negative
PRRT1	protein_coding	AK055458	noncoding	-0.999877	4.65E-12	negative
NCAPG	protein_coding	BC040735	noncoding	-0.99987019	5.47E-12	negative
LILRB4	protein_coding	C1QTNF3-AMACR	noncoding	-0.99986481	6.18E-12	negative
DEFA4	protein_coding	AK055811	noncoding	-0.99986385	6.31E-12	negative
GRAMD1A	protein_coding	BC017578	noncoding	-0.9998579	7.17E-12	negative
ZNF398	protein_coding	RP11-44F14.5	noncoding	-0.99985766	7.21E-12	negative
SPSB4	protein_coding	CHEK2P2	noncoding	-0.99985489	7.64E-12	negative
KLKB1	protein_coding	C15orf45	noncoding	-0.99984634	9.07E-12	negative
BBC3	protein_coding	AC000067.1	noncoding	-0.99983956	1.03E-11	negative
SEMA3F	protein_coding	BC044655	noncoding	-0.99983854	1.05E-11	negative
KDM5B	protein_coding	HMlincRNA1639	noncoding	-0.99983141	1.20E-11	negative
SKA1	protein_coding	BC040735	noncoding	-0.99982305	1.39E-11	negative
SBF2	protein_coding	AK055811	noncoding	-0.99982105	1.43E-11	negative
TIMP4	protein_coding	LINC00514	noncoding	-0.99981752	1.52E-11	negative
NR6A1	protein_coding	LINC00461	noncoding	-0.99981217	1.66E-11	negative

PDLIM5	protein_coding	AC004383.4	noncoding	-0.99981105	1.69E-11	negative
SGSM3	protein_coding	RP11-406O23.2	noncoding	-0.99981061	1.70E-11	negative
PLA2G16	protein_coding	KRT18P55	noncoding	-0.99981035	1.71E-11	negative
CD300LG	protein_coding	C6orf147	noncoding	-0.99980777	1.78E-11	negative
NREP	protein_coding	BANCR	noncoding	0.999800259	1.99E-11	positive
TRIM36	protein_coding	LINC00304	noncoding	0.999800582	1.98E-11	positive
HNRNPA1	protein_coding	GUSBP9	noncoding	0.999800903	1.97E-11	positive
TMEM45A	protein_coding	AC127904.2	noncoding	0.999801224	1.96E-11	positive
RPL41	protein_coding	H1FX-AS1	noncoding	0.999801343	1.96E-11	positive
NEK3	protein_coding	BC107568	noncoding	0.999801534	1.95E-11	positive
RRM2	protein_coding	RP11-119F7.5	noncoding	0.999802246	1.93E-11	positive
IRX2	protein_coding	AX746605	noncoding	0.999803302	1.90E-11	positive
KDM1A	protein_coding	AC034193.5	noncoding	0.999804104	1.88E-11	positive
SPIN4	protein_coding	LOC100505806	noncoding	0.999804328	1.87E-11	positive
COIL	protein_coding	BC045725	noncoding	0.999804615	1.86E-11	positive
SPC25	protein_coding	RP11-119F7.5	noncoding	0.999805011	1.85E-11	positive
NTS	protein_coding	AC002456.2	noncoding	0.999805103	1.85E-11	positive
BEX1	protein_coding	BC044655	noncoding	0.999805111	1.85E-11	positive
CNRIP1	protein_coding	AK124433	noncoding	0.999805585	1.84E-11	positive
NPPB	protein_coding	DLEU2	noncoding	0.999807622	1.78E-11	positive
SBF2	protein_coding	RP11-395I6.3	noncoding	0.999807758	1.78E-11	positive
STOM	protein_coding	CTD-2116N20.1	noncoding	0.999807951	1.77E-11	positive
SPIRE1	protein_coding	LRRC37A4	noncoding	0.99980801	1.77E-11	positive
NLE1	protein_coding	CTD-3110H11.2	noncoding	0.999808043	1.77E-11	positive
NEK2	protein_coding	CTD-3080F16.3	noncoding	0.999808083	1.77E-11	positive
TBX20	protein_coding	AB074166	noncoding	0.999808423	1.76E-11	positive
HBG1	protein_coding	HBBP1	noncoding	0.999808491	1.76E-11	positive
SLC2A1	protein_coding	LOC100506123	noncoding	0.999808498	1.76E-11	positive
PABPC4L	protein_coding	LOC100505806	noncoding	0.999809115	1.74E-11	positive
ZDHHC17	protein_coding	XLOC_008100	noncoding	0.999809158	1.74E-11	positive
DEK	protein_coding	BC043001	noncoding	0.999809475	1.73E-11	positive
STARD4	protein_coding	AP000692.9	noncoding	0.999809943	1.72E-11	positive
RNASEH2A	protein_coding	BC042823	noncoding	0.999810803	1.69E-11	positive
CENPW	protein_coding	BANCR	noncoding	0.999811676	1.67E-11	positive
KDELR1	protein_coding	AF075112	noncoding	0.999811962	1.66E-11	positive
CHML	protein_coding	ANKRD36BP2	noncoding	0.999812937	1.64E-11	positive
XPNPEP1	protein_coding	LOC678655	noncoding	0.999813106	1.63E-11	positive
RAD51	protein_coding	AX748379	noncoding	0.999813257	1.63E-11	positive
CDK6	protein_coding	BC017578	noncoding	0.999813728	1.62E-11	positive
TOP2A	protein_coding	AX747860	noncoding	0.999814553	1.59E-11	positive
IRX2	protein_coding	CTD-2270L9.4	noncoding	0.999815367	1.57E-11	positive
KCNH7	protein_coding	AK098438	noncoding	0.999815813	1.56E-11	positive
HBG2	protein_coding	AX747860	noncoding	0.999815923	1.56E-11	positive
TRO	protein_coding	RP5-901A4.1	noncoding	0.999817029	1.53E-11	positive
MSL2	protein_coding	FLJ39051	noncoding	0.999817405	1.52E-11	positive
NET1	protein_coding	AK094859	noncoding	0.999817524	1.52E-11	positive
FOLH1	protein_coding	AC127904.2	noncoding	0.999817566	1.52E-11	positive
NREP	protein_coding	LOC100129617	noncoding	0.999817786	1.51E-11	positive
NPPA	protein_coding	HHIP-AS1	noncoding	0.999818072	1.51E-11	positive
SOWAHC	protein_coding	AK098438	noncoding	0.999819611	1.47E-11	positive
VEGFA	protein_coding	ACTN1-AS1	noncoding	0.999819788	1.46E-11	positive
GRK5	protein_coding	ACTN1-AS1	noncoding	0.999820101	1.46E-11	positive
MCM10	protein_coding	BANCR	noncoding	0.999820167	1.45E-11	positive
GJA1	protein_coding	AX746605	noncoding	0.999820354	1.45E-11	positive
C7orf29	protein_coding	AGSK1	noncoding	0.999820408	1.45E-11	positive
MIXL1	protein_coding	LINC00304	noncoding	0.99982051	1.45E-11	positive

SNX7	protein_coding	BC044655	noncoding	0.999820719	1.44E-11	positive
H3F3B	protein_coding	BANCR	noncoding	0.999821135	1.43E-11	positive
STAG2	protein_coding	RP11-105C19.1	noncoding	0.9998217	1.42E-11	positive
TMSB15A	protein_coding	RP11-389O22.1	noncoding	0.999821929	1.41E-11	positive
NDUFA7	protein_coding	BC100777	noncoding	0.99982195	1.41E-11	positive
NET1	protein_coding	C6orf147	noncoding	0.999822584	1.40E-11	positive
CLDN3	protein_coding	BC043001	noncoding	0.999823702	1.37E-11	positive
SPIN4	protein_coding	RP11-406O23.2	noncoding	0.999824225	1.36E-11	positive
RBM24	protein_coding	AX746967	noncoding	0.999824229	1.36E-11	positive
TMEM200C	protein_coding	SNHG8	noncoding	0.999824531	1.35E-11	positive
LOC81691	protein_coding	HBBP1	noncoding	0.999824553	1.35E-11	positive
HMGN5	protein_coding	AX748120	noncoding	0.999824735	1.35E-11	positive
NDC80	protein_coding	LOC100506123	noncoding	0.999824768	1.35E-11	positive
RAB40AL	protein_coding	AL833634	noncoding	0.999824838	1.34E-11	positive
CDS1	protein_coding	AC127904.2	noncoding	0.999824928	1.34E-11	positive
CDC45	protein_coding	AX747860	noncoding	0.99982555	1.33E-11	positive
CDK6	protein_coding	AL833346	noncoding	0.999825804	1.32E-11	positive
PF4V1	protein_coding	AC127904.2	noncoding	0.99982602	1.32E-11	positive
NPPB	protein_coding	NOP14-AS1	noncoding	0.999826808	1.30E-11	positive
KIAA0101	protein_coding	AC034193.5	noncoding	0.999827198	1.29E-11	positive
PHF19	protein_coding	HBBP1	noncoding	0.999827199	1.29E-11	positive
SPIRE1	protein_coding	AC002456.2	noncoding	0.999827207	1.29E-11	positive
HDAC1	protein_coding	AX721280	noncoding	0.999827349	1.29E-11	positive
C6orf25	protein_coding	AE000661.37	noncoding	0.99982893	1.25E-11	positive
MCM10	protein_coding	HBBP1	noncoding	0.999828942	1.25E-11	positive
TWSG1	protein_coding	AC006028.9	noncoding	0.999829273	1.24E-11	positive
TOP2A	protein_coding	LOC145474	noncoding	0.999829391	1.24E-11	positive
RPL7L1	protein_coding	CTC-367J11.1	noncoding	0.999829573	1.24E-11	positive
TMEM45A	protein_coding	AK094859	noncoding	0.99983022	1.22E-11	positive
PCDH18	protein_coding	BC043001	noncoding	0.999831486	1.20E-11	positive
FOXS1	protein_coding	FLJ42875	noncoding	0.999831895	1.19E-11	positive
TYMS	protein_coding	AC127904.2	noncoding	0.999832048	1.18E-11	positive
EDDM3B	protein_coding	CTC-454M9.1	noncoding	0.99983238	1.18E-11	positive
TNKS	protein_coding	C12orf32	noncoding	0.999832538	1.17E-11	positive
NOVA1	protein_coding	FAS	noncoding	0.999832733	1.17E-11	positive
CAMP	protein_coding	AL833346	noncoding	0.999832862	1.17E-11	positive
GPR39	protein_coding	AX747860	noncoding	0.999832969	1.16E-11	positive
PDCD4	protein_coding	AX747826	noncoding	0.999834543	1.13E-11	positive
TNNI1	protein_coding	PRKY	noncoding	0.999834977	1.12E-11	positive
P2RY14	protein_coding	AC093609.1	noncoding	0.999835925	1.10E-11	positive
CLSPN	protein_coding	BC043001	noncoding	0.999836239	1.10E-11	positive
TMEM45A	protein_coding	BC044655	noncoding	0.999837336	1.08E-11	positive
ZNF292	protein_coding	RP11-69I8.3	noncoding	0.999837362	1.08E-11	positive
SQLE	protein_coding	RP11-706O15.3	noncoding	0.999837622	1.07E-11	positive
RACGAP1	protein_coding	CTC-340A15.2	noncoding	0.999837836	1.07E-11	positive
SPC25	protein_coding	LOC400794	noncoding	0.99983899	1.04E-11	positive
HIST1H3F	protein_coding	BC043001	noncoding	0.999839851	1.03E-11	positive
TMSB15A	protein_coding	RP11-119F7.5	noncoding	0.999840361	1.02E-11	positive
H3F3B	protein_coding	AX747860	noncoding	0.999841112	1.00E-11	positive
NAALAD2	protein_coding	AC004069.2	noncoding	0.999841228	1.00E-11	positive
ZNF726	protein_coding	RP1-315G1.3	noncoding	0.999841612	9.93E-12	positive
DEPDC1	protein_coding	AC034193.5	noncoding	0.999841619	9.93E-12	positive
KRTDAP	protein_coding	ACTN1-AS1	noncoding	0.999841987	9.86E-12	positive
CCNG1	protein_coding	AC004540.4	noncoding	0.999842003	9.86E-12	positive
RPL23A	protein_coding	AK023507	noncoding	0.99984201	9.86E-12	positive
SRPX	protein_coding	FAS	noncoding	0.999842805	9.71E-12	positive

SEPT10	protein_coding	RP11-395I6.3	noncoding	0.999843364	9.61E-12	positive
SLC2A1	protein_coding	RP11-368I23.2	noncoding	0.999845197	9.27E-12	positive
LNX2	protein_coding	BC044655	noncoding	0.999846468	9.05E-12	positive
OIP5	protein_coding	AK025321	noncoding	0.999847182	8.92E-12	positive
XPO1	protein_coding	AF001548.5	noncoding	0.999848181	8.75E-12	positive
NCAPG	protein_coding	AX747860	noncoding	0.999848421	8.71E-12	positive
GP6	protein_coding	AC003075.4	noncoding	0.999849303	8.55E-12	positive
MCM10	protein_coding	AX747860	noncoding	0.999851209	8.23E-12	positive
MST1	protein_coding	AC002456.2	noncoding	0.999852167	8.08E-12	positive
C7orf29	protein_coding	ALMS1P	noncoding	0.999852742	7.98E-12	positive
BIRC5	protein_coding	AX747860	noncoding	0.999852797	7.97E-12	positive
SPINT2	protein_coding	RP5-821D11.7	noncoding	0.999853033	7.93E-12	positive
KIDINS220	protein_coding	CR936711	noncoding	0.999853141	7.92E-12	positive
C15orf59	protein_coding	BX004987.5	noncoding	0.999854034	7.77E-12	positive
TRIM36	protein_coding	RP5-916O11.2	noncoding	0.999854211	7.75E-12	positive
LECT1	protein_coding	AX747860	noncoding	0.999854213	7.75E-12	positive
TIA1	protein_coding	AX746640	noncoding	0.999854645	7.68E-12	positive
SPIRE1	protein_coding	AK131521	noncoding	0.999855192	7.59E-12	positive
NUDT5	protein_coding	AC129778.2	noncoding	0.999855448	7.55E-12	positive
S100A9	protein_coding	LINC00501	noncoding	0.999855597	7.53E-12	positive
TTK	protein_coding	DQ595103	noncoding	0.999857174	7.28E-12	positive
FAM5C	protein_coding	CAHM	noncoding	0.999858106	7.14E-12	positive
MCM10	protein_coding	AC034193.5	noncoding	0.999858588	7.07E-12	positive
CNTROB	protein_coding	CHEK2P2	noncoding	0.999859373	6.95E-12	positive
POU3F2	protein_coding	LINC00514	noncoding	0.999860505	6.79E-12	positive
PPP1R14C	protein_coding	LOC284080	noncoding	0.999860917	6.73E-12	positive
NOVA1	protein_coding	AK123878	noncoding	0.99986213	6.55E-12	positive
RPL7L1	protein_coding	PLAC2	noncoding	0.999862718	6.47E-12	positive
PRC1	protein_coding	HBBP1	noncoding	0.999862786	6.46E-12	positive
COL1A2	protein_coding	AL592284.1	noncoding	0.999864212	6.26E-12	positive
CLK1	protein_coding	AC012074.2	noncoding	0.99986425	6.25E-12	positive
FGF18	protein_coding	C17orf76-AS1	noncoding	0.999864436	6.23E-12	positive
SCNN1B	protein_coding	AE000661.37	noncoding	0.999865089	6.14E-12	positive
TTC32	protein_coding	RP11-315O6.1	noncoding	0.999865205	6.12E-12	positive
LXN	protein_coding	LOC100272217	noncoding	0.999865498	6.08E-12	positive
SHCBP1L	protein_coding	RP11-328C8.5	noncoding	0.999866608	5.93E-12	positive
CACNB2	protein_coding	AK131521	noncoding	0.999867372	5.83E-12	positive
GPR39	protein_coding	AC034193.5	noncoding	0.999867816	5.77E-12	positive
CEP78	protein_coding	BC037304	noncoding	0.999868314	5.71E-12	positive
CACNB2	protein_coding	AC127904.2	noncoding	0.999868853	5.64E-12	positive
PPBP	protein_coding	LOC84989	noncoding	0.99986973	5.53E-12	positive
BEX1	protein_coding	AC012074.2	noncoding	0.999871117	5.35E-12	positive
NRAS	protein_coding	LOC646762	noncoding	0.99987165	5.29E-12	positive
OIP5	protein_coding	CR936711	noncoding	0.999871729	5.28E-12	positive
SHCBP1L	protein_coding	AK023507	noncoding	0.999872938	5.13E-12	positive
GID8	protein_coding	CTC-367J11.1	noncoding	0.99987406	4.99E-12	positive
TMEM133	protein_coding	AK023507	noncoding	0.999874677	4.92E-12	positive
SECISBP2L	protein_coding	KIAA0664L3	noncoding	0.999874786	4.91E-12	positive
SPIRE1	protein_coding	AK094859	noncoding	0.999875728	4.80E-12	positive
SERPINF1	protein_coding	RP11-1021N1.2	noncoding	0.999876397	4.72E-12	positive
PTTG1	protein_coding	LINC00482	noncoding	0.999877151	4.63E-12	positive
PDCD7	protein_coding	DLEU2	noncoding	0.999877894	4.55E-12	positive
TMEM45A	protein_coding	RP11-389O22.1	noncoding	0.999879071	4.42E-12	positive
TSPAN5	protein_coding	RP11-291L15.2	noncoding	0.999879276	4.40E-12	positive
ACYP1	protein_coding	AC078883.3	noncoding	0.999881371	4.17E-12	positive
KDM1A	protein_coding	HBBP1	noncoding	0.999881526	4.16E-12	positive

NET1	protein_coding	BC044655	noncoding	0.999881542	4.16E-12	positive
TMSB15A	protein_coding	LOC100505806	noncoding	0.999882536	4.05E-12	positive
VEGFA	protein_coding	DLEU2	noncoding	0.999883461	3.96E-12	positive
SKA2	protein_coding	RP11-640N20.6	noncoding	0.999883733	3.93E-12	positive
RPL23A	protein_coding	RP11-328C8.5	noncoding	0.999883762	3.93E-12	positive
BIRC5	protein_coding	AC034193.5	noncoding	0.999883995	3.90E-12	positive
COL3A1	protein_coding	CHEK2P2	noncoding	0.999886364	3.67E-12	positive
RPL23A	protein_coding	BC017578	noncoding	0.999886755	3.63E-12	positive
TRO	protein_coding	AC046143.7	noncoding	0.999887222	3.59E-12	positive
TOP2A	protein_coding	BANCR	noncoding	0.999887457	3.56E-12	positive
PRIM1	protein_coding	AC005009.1	noncoding	0.999888302	3.48E-12	positive
HIST4H4	protein_coding	AC005592.1	noncoding	0.999889464	3.38E-12	positive
RRM1	protein_coding	LRRC37A4	noncoding	0.999891978	3.15E-12	positive
HSPA1B	protein_coding	DQ596274	noncoding	0.999892763	3.08E-12	positive
MTF2	protein_coding	BC044655	noncoding	0.999895099	2.89E-12	positive
C11orf82	protein_coding	BC107568	noncoding	0.999895153	2.88E-12	positive
CACNB2	protein_coding	AK094859	noncoding	0.99989594	2.82E-12	positive
KIDINS220	protein_coding	AK025321	noncoding	0.999896543	2.77E-12	positive
TBC1D4	protein_coding	RP1-163G9.1	noncoding	0.999896882	2.74E-12	positive
CHEK2	protein_coding	AC127904.2	noncoding	0.999897743	2.67E-12	positive
ITSN2	protein_coding	CLEC2D	noncoding	0.999898152	2.64E-12	positive
SPIN4	protein_coding	AC004383.4	noncoding	0.999898677	2.60E-12	positive
FOS	protein_coding	CTC-426B10.1	noncoding	0.999899885	2.51E-12	positive
CAMP	protein_coding	BC017578	noncoding	0.999900788	2.44E-12	positive
VCX3A	protein_coding	RP11-849N15.2	noncoding	0.999901566	2.38E-12	positive
PCNXL4	protein_coding	CCDC144C	noncoding	0.999902173	2.34E-12	positive
EDDM3B	protein_coding	AX748120	noncoding	0.99990252	2.32E-12	positive
NOTCH2NL	protein_coding	AX747913	noncoding	0.999905659	2.10E-12	positive
PRC1	protein_coding	AC034193.5	noncoding	0.999907227	2.00E-12	positive
TRIM36	protein_coding	TMPO-AS1	noncoding	0.999907786	1.96E-12	positive
C17orf96	protein_coding	BC044655	noncoding	0.999909483	1.85E-12	positive
KIAA0101	protein_coding	HBBP1	noncoding	0.99990955	1.85E-12	positive
TUBB3	protein_coding	DLEU2	noncoding	0.999911635	1.72E-12	positive
PCNXL4	protein_coding	LOC100505815	noncoding	0.999912094	1.70E-12	positive
GID8	protein_coding	AC004540.4	noncoding	0.999912442	1.68E-12	positive
OR2B6	protein_coding	LDHA	noncoding	0.999913615	1.61E-12	positive
TRIM36	protein_coding	AX747860	noncoding	0.999914439	1.57E-12	positive
MIXL1	protein_coding	AX747860	noncoding	0.999914917	1.54E-12	positive
SPIN4	protein_coding	RP11-119F7.5	noncoding	0.999915634	1.50E-12	positive
C1orf112	protein_coding	C12orf32	noncoding	0.999917154	1.42E-12	positive
CDC42EP3	protein_coding	C6orf147	noncoding	0.999920382	1.26E-12	positive
DEFA3	protein_coding	AC004383.4	noncoding	0.999923221	1.13E-12	positive
SLC2A1	protein_coding	CR936711	noncoding	0.999929329	8.82E-13	positive
BUB1	protein_coding	AC034193.5	noncoding	0.999932347	7.74E-13	positive
RDX	protein_coding	HMGB3P22	noncoding	0.99993572	6.64E-13	positive
NAALAD2	protein_coding	AC069277.2	noncoding	0.999940563	5.25E-13	positive
ALB	protein_coding	AC004383.4	noncoding	0.999943424	4.53E-13	positive
CENPF	protein_coding	AX747860	noncoding	0.999943497	4.51E-13	positive
CARD18	protein_coding	AC116035.1	noncoding	0.999945764	3.99E-13	positive
FAM69C	protein_coding	BC107568	noncoding	0.999949498	3.22E-13	positive
SNCA	protein_coding	ACTN1-AS1	noncoding	0.999950726	2.99E-13	positive
LCORL	protein_coding	CHEK2P2	noncoding	0.999951181	2.91E-13	positive
PTTG1	protein_coding	AC004383.4	noncoding	0.999951718	2.81E-13	positive
COL1A1	protein_coding	AX747860	noncoding	0.999958271	1.82E-13	positive
MYOCD	protein_coding	AGSK1	noncoding	0.999965053	1.07E-13	positive
